# Response to Wu et al. — Cost-effectiveness analysis of infant pneumococcal vaccination in Malaysia and Hong Kong

**DOI:** 10.1080/21645515.2016.1192738

**Published:** 2016-07-26

**Authors:** Lijoy Varghese, Bruce Mungall, Xu-Hao Zhang, Bernard Hoet

**Affiliations:** a GSK Vaccines, Singapore, Singapore; b GSK Vaccines, Wavre, Belgium

**Keywords:** herd effect, pneumococcal conjugate vaccine (PCV), nasopharyngeal carriage, cross-protection, non-typeable *Haemophilus influenzae* (NT*Hi*), 13-valent pneumococcal conjugate vaccine (PCV13), 10-valent pneumococcal non-typeable *Haemophilus influenzae* protein D conjugate vaccine (PHiD-CV)

## Abstract

A recently published paper that assessed the comparative cost-effectiveness of the 2 pneumococcal conjugate vaccines (PCVs) in Malaysia and Hong Kong reported that the 13-valent PCV vaccine (PCV13) is a better choice compared to the 10-valent pneumococcal non-typeable *Haemophilus influenzae* protein D conjugate vaccine (PHiD-CV or PCV10) from both a payer and societal perspective as well as under various scenarios. However, the analysis relied on a large number of assumptions that were either erroneous or did not take into account the most recent body of evidence available. A rigorous evaluation of the underlying assumptions is necessary to present a fair and balanced analysis for decision-making.

## Introduction

Wu et al. have presented a cost-effectiveness analysis by evaluating the clinical and economic benefits of a routine vaccination program in Malaysia and Hong Kong using the available 10-valent pneumococcal non-typeable *Haemophilus influenzae* protein D conjugate vaccine (PHiD-CV; *Synflorix*™) and 13-valent pneumococcal conjugate vaccine (PCV13; *Prevenar 13*).^1^ While the modeling methodologies are rigorous and sound, many of the assumptions made are inconsistent with the most current published scientific evidence. Key among the erroneous assumptions include the rationale for not applying any measure of herd effects for PHiD-CV, assuming no cross-protection for serotypes 6A and 19A for PHiD-CV as well as assuming no impact of PHiD-CV on non-typeable *Haemophilus influenzae* acute otitis media (NT*Hi* AOM). These assumptions have vastly over-estimated the cost-effectiveness results obtained by the authors with regard to PCV13 over PHiD-CV in both countries.

## Nasopharyngeal carriage and herd effect

Wu et al. have stated that herd-effects from vaccination play a significant role in the cost-effectiveness of pneumococcal conjugate vaccines (PCVs).^1^ This is feasible because pneumococcal vaccination in communities has been consistently followed by significant decreases in both vaccine-type (VT)-carriage and VT-invasive pneumococcal disease (IPD) in unvaccinated groups. While the magnitudes of the decreases may not be congruent, even in communities which reported the smallest ratio of VT-IPD decline to VT-carriage decline, the decrease in IPD represents a significant public health gain.^2^

A meta-analysis of randomized control trials (RCTs) that looked at the impact of PCVs on nasopharyngeal carriage (NPC) in the population targeted by vaccination demonstrated a reduction in carriage for VT pneumococcus compared to no vaccination with a relative risk (RR) of NPC of 0.67 (95% confidence interval [CI]: 0.56, 0.81).^3^ However, the benefits from reduction in VT-disease also have to be considered in the context of serotype replacement. The meta-analysis also reported that non-VT-carriage increased in line with the theory of serotype replacement with a RR of 1.23 (95% CI: 1.09, 1.40).^3^ Consequently, the overall impact on carriage was statistically inconclusive with a RR of 0.96 (95% CI: 0.91, 1.01).^3^

Another review by Davis et al. also demonstrated a similar trend from 14 observational studies: VT-IPD and VT-NPC showed decreases in the age-groups not targeted for vaccination.^2^ This review, however, reported moderate decreases in all-type IPD in the older age groups.^2^ It must also be noted that the majority of the studies that looked at this outcome reported the incidence rates of specific diseases over a single year post-vaccination. The authors also state that decreases in VT-NPC is not an “ideal proxy” for the indirect impact of the PCVs, but possibly one of the many factors that influence it.^2^

The study that Wu et al. have referenced to demonstrate the impact of PCV13 on NPC in children with AOM does not conclusively demonstrate the effect.^4^ The authors in that study observed a reduction in NPC of the 6 additional serotypes covered in PCV13 (but not in the 7-valent PCV, PCV7), but a closer evaluation of the data shows a significant effect only for serotypes 7F and 19A, but not for the other 4 serotypes (1, 3, 5 and 6A).^4^

When considering the herd impact of PCV13 in the United Kingdom (UK) surveillance system, while the absolute number of cases of IPD caused by serotypes included in PCV13 (but not in PCV7) in people ≥65 years of age decreased from 2010/11 to 2013/14, there was an uncharacteristic and substantial increase in cases in 2014/15 despite high vaccine coverage.^5^ The herd impact becomes significantly less clear when considering the IPD cases in the same age group caused by serotypes not included in PCV13.^6^ The number of those cases increased from approximately 1,000 in 2009/10 to approximately 1,800 in 2014/15 which offsets the decrease in IPD cases from the earlier category almost 2-fold.^6^

Wu et al. have also further stated that at the time of publication (submitted on 03 March, 2015; and revised on 19 May, 2015) there were no data to support the indirect impact of PHiD-CV on pneumococcal diseases in adults;^1^ this being the premise of their decision to not incorporate any measure of indirect vaccine impact for the vaccine. However, the recent body of published evidence points to the contrary. Jokinen et al. showed that in the Finnish Invasive Pneumococcal disease (FinIP) vaccine trial, PHiD-CV demonstrated an efficacy against VT-NPC of 29% (95% CI: 6, 47) in the siblings (aged 3 to 7 years) of the vaccinated children.^7^ If one is to be consistent with the assumptions of Wu et al. – that a reduction in NPC could be used as a proxy for the indirect impact on the non-vaccinated population, – then PHiD-CV is also expected to demonstrate a significant indirect effect in the unvaccinated population. This assumption can be substantiated from the results of a population-based observational study in Finland by Jokinen et al., which reported a 48% (95% CI: 18, 69) reduction in IPD among unvaccinated children aged 2 to 5 years with PHiD-CV.^8^

Additionally, publically available surveillance data from a number of countries clearly demonstrate herd effects of PHiD-CV in older adults following the introduction of childhood vaccination programs in Finland,^9^ New Zealand^10^ and in the Quebec province in Canada^11^ wherein VT herd-effects were observed in all ages.

Thus, considering the available evidence and the impact of herd effects on the final results reported by Wu et al., assuming herd protection for PCV13 alone is, in our opinion, not an objective assumption.

## Cross-protection for non-vaccine serotypes

Wu et al. also have claimed that an earlier study on the cost-effectiveness of PHiD-CV compared to PCV13 conducted in Malaysia^12^ “*relied on a number of questionable assumptions favoring PCV10 that lacked scientific validation*.”^1^ The authors pointed to, among others, the assumption of cross-protection for PHiD-CV against serotypes 6A and 19A.^1^ However, data to support the assumption of cross-protection for PHiD-CV is presented in a number of robustly designed studies from Brazil,^13^ Canada^14^ and Finland.^8^

The authors claim that the results of the case-control study in Brazil “*are inconsistent with the national surveillance system in Brazil, which shows an increase in the incidence of serotype 19A invasive pneumococcal disease between 2006 and 2011 in children younger than 5 y[ears] of age*.”^1^ However, the references^15,16^ cited by the authors explain the reason why such a comparison is flawed. Firstly, while a lab-based passive surveillance that captures only a fraction of IPD cases is suitable for evaluating trends in serotype distribution and antibiotic resistance, it is not an ideal system for performing a quantitative assessment of vaccine effectiveness. Secondly, a simple comparison between the numbers reported between 2006 and 2011 could be biased by changes in the sensitivity of surveillance procedures and a switch toward active surveillance introduced as a part of the case-control vaccine effectiveness study conducted between 2010 and 2012.^13^ Lastly, PHiD-CV was introduced into the national immunization program (NIP) in Brazil only in June 2010.^16^ The reported coverage in children <1 year of age was 81.5% in 2011^16^ and the proportion of 19A in the serotyped IPD cases was 2.2%^16^ compared to 7% in 2006^15^ (albeit in children <5 years of age; the only data readily available). A cross-sectional study that looked at vaccination coverage in the municipality of Goiana in mid-western Brazil from December 2010 to February 2011 found that overall vaccination coverage was 53.4% compared to the diphtheria, tetanus, pertussis-*Haemophilus influenzae* type B (DTP-Hib) vaccine coverage of 93% and compliance with the recommended schedules was only 16.6%.^17^

The 82% (95% CI: 11, 96) effectiveness of PHiD-CV against 19A IPD reported in the Brazilian case–control study^13^ has been corroborated by 2 additional robustly designed studies in Quebec (Canada)^14^ and Finland^8^. In the Quebec case–control study, the effectiveness of PHiD-CV against serotype 19A was 71% (95% CI: 24, 89).^14^ This study, which is unique in also assessing the effectiveness of PCV13 on 19A disease in the same study setting, demonstrated vaccine effectiveness of 74% (95% CI: 11, 92) for PCV13 (overlapping confidence intervals with PHiD-CV).^14^ In the Finnish cohort study, a significant 62% (95% CI: 20, 85) reduction in a number of 19A IPD cases was observed following the introduction of PHiD-CV into their NIP.^8^ Additional data supporting the effectiveness of PHiD-CV into NIPs, including that of the Netherlands,^18^ as well as the functional opsonophagocytic antibody (OPA) responses against 19A (functional OPAs are generally agreed to be the mechanism of protection) have been fully described in recent reviews by Hausdorff et al.^19^ and Mrkvan et al.^20^

Evidence for cross-protection of PHID-CV against IPD caused by serotype 6A is less uniform, but the data generally indicate a positive impact. In the Quebec case–control study, the effectiveness of PHiD-CV against the 10 serotypes included in the vaccine and 6A was 97% (95% CI: 84, 99).^14^ In the Finnish cohort study, the relative rate reduction for PHiD-CV against serotype 6A was 100% (95% CI: 41, 100).^8^ However, the Brazilian case–control study presented a much lower non-significant value of 14.7% (95% CI: −311.6, 82.3) although the authors mention that a possible reason for low estimate is due to the small number of cases of 6A disease.^13^

While the Quebec and Finnish studies were published around the same time Wu et al. submitted their revised manuscript, the Brazil data was available prior to this date and should have been considered regarding cross-protection data for PHiD-CV.

## Effectiveness against all-cause pneumonia

Wu et al. have assumed vaccine effectiveness against all cause pneumonia for either vaccine based on the number of serotypes covered in the vaccine and the serotype distribution in the corresponding country.^1^ Hausdorff et al. explain why serotype-based approach to estimate vaccine effectiveness for PCVs is a flawed one to model disease impact^21^ and it is clear from the discussion above that this simplistic approach is not suitable because it does not account protection from disease due to cross-reactive serotypes. This approach is even more unsuitable for the more nuanced cases of all-cause pneumonia and AOM for which the causative pathogens are often not known. A systematic review of RCTs by Lucero et al. that assessed the efficacy of PCVs against pneumonia found no conclusive evidence that higher valent vaccines offer greater protection against clinical or radiologically-confirmed pneumonia.^22^ While evidence for protection against all-cause pneumonia for PHiD-CV was obtained from 2 RCTs^23,24^ and corroborated during post-marketing surveillance,^25,26^ evidence for PCV13, on the other hand, comes primarily from post-marketing surveillance studies. Comparing data across different post-marketing surveillance studies is difficult due to the large number of confounders not accounted for in the study designs and any inferences thus made are inherently biased. This can be observed from a recent study in Sweden by Berglund et al.^27^ That study looked at different county councils in Sweden that used PCV7 before switching to either PCV13 or PHiD-CV. The authors conclude that the difference in the rates of hospitalization from all-cause pneumonia between the 2 groups can be attributed to the difference in the valence of the vaccines used.^27^ However, a closer look at the data shows that even in the period where the 2 groups of county councils used PCV7, the rates of all-cause pneumonia hospitalization are markedly different despite the demographic and socio-economic status similarities; implying the presence of a confounding factor.^27^ Other flaws in the study design, such as the lack of consideration of a significant transition period between vaccines, also complicate the ability to make any inference between the 2 groups. The authors report a reduction of 37% (incidence rate ratio [IRR] 0.63; 95% CI: 0.54, 0.74) in the risk of hospitalization in counties that used PCV7 followed by PCV13 compared to the pre-PCV period.^27^ However, another study performed in Stockholm, Sweden during the same period by Lindstrand et al. presents a very different impact of PCV7/PCV13 compared to the pre-PCV period: a 19% reduction (IRR 0.81; 95% CI: 0.74–0.89).^28^ These results are not dissimilar to the PHiD-CV efficacy values against consolidated community-acquired pneumonia: 21.8% (95% CI: 7.7, 33.7).^23^

Thus, given the lack of comparability across post-marketing studies and the body of evidence that shows no additional impact of higher-valent vaccines on all-cause pneumonia, both PHiD-CV and PCV13 can be assumed to offer similar protection.

## Impact on NTHi AOM

Wu et al. have stated that “*there is no evidence to support an impact of PCV10 on otitis media greater than that expected from the pneumococcal serotypes contained in the vaccine.*”^1^ Again, the available evidence on the impact of PCVs on NT*Hi* AOM is more nuanced than the authors state.

The Finnish Otitis Media (FinOM) Vaccine Trial showed a non-significant impact of 6% (95% CI: −4, 16) for PCV7 against all-cause AOM.^29^ The positive impact of the vaccine of 34% (95% CI: 21, 45) against culture confirmed pneumococcus was partially offset by the negative impact of −11% (95% CI: −34, 8) against AOM caused by *Haemophilus influenzae*.^29^ The data obtained from the study is in line with those in the United States (US) study^30^ referenced by Wu et al. That study demonstrated a vaccine efficacy against otitis media visits of 7.8% (95% CI: 5.2, 10.5).^30^ In France, pediatricians who participated in a prospective NPC study since 2001 as a part of the country-wide Association Clinique Thérapeutique Infantile du Val de Marne (ACTIV) network that looked at children with suppurative AOM with fever and/or otalgia observed a slight but significant decrease in overall pneumococcal carriage (−15%, 71.2% in 2001 to 56.2% in 2014) due to the introduction of PCV7 followed by PCV13.^31^ The authors also reported that during the period, the carriage of some non-VT serotypes increased.^31^ Additionally, in the middle-ear fluid (MEF) obtained from children with AOM who did not respond to antibiotic treatment, *Haemophilus influenzae* emerged as the most significant pathogen, from 39.2% before PCV7 introduction (1996-98) to 75.9% following PCV13 implementation.^31^ This pattern seems to corroborate the findings from the earlier FinOM study of serotype and pathogen replacement.

PHiD-CV, on the other hand, was shown to have a significant positive impact of 19% (95% CI: 4.4, 31.4) on all-cause AOM.^23^ More importantly, the vaccine was demonstrated to have a positive, albeit non-significant, impact of 17.3% (95% CI: −49.8, 54.3) against AOM caused by *Haemophilus influenzae*.^23^ This trend was also observed in the pre-cursor vaccine 11-valent pneumococcal protein D conjugate vaccine (11Pn-PD) with an efficacy of 35.6% (95% CI: 3.8, 57.0) against AOM caused by *Haemophilus influenzae*.^32^

In a study that looked at the NPC and middle-ear discharge (ED) microbiology in vaccinated indigenous children in Australia, the prevalence of NT*Hi*-infected ED was observed to be lower in PHiD-CV vaccinated children (34%) compared to PCV7 vaccinated children (61%).^33^ Concurrently, there was no substantial difference in the serotypes colonising the nasopharynx of PCV7 compared to PHiD-CV vaccinated children.^33^ The authors hypothesized that vaccine-induced immune responses could deliver protection in the middle ear, where numbers of organisms are likely to be lower, without eliminating NPC.^33^

In light of the evidence presented here, we believe that it is erroneous to use the highly simplified serotype coverage based approach to estimating the vaccine efficacy of the PCVs against AOM and also to attribute no efficacy against NT*Hi* AOM for PHiD-CV.

## Serotype 3 protection

Wu et al. have claimed that “*there have not been any conclusive evidence of the lack of PCV13 effectiveness against serotype 3*.”^1^ Despite the several years after introduction of PCV13 into many NIPs, there is still no conclusive evidence that PCV13 confers the same level of protection against IPD caused by serotype 3 as it does for the other serotypes included in the vaccine or that it provides herd protection.^34-41^ Although the latest Joint Committee on Vaccines and Immunization (JCVI) minutes from the UK have reported non-significant trends for reduction,^42^ it is unclear whether these observations should be attributed to the use of the vaccine, or instead represent a secular trend/natural cyclical pattern in the disease, as have been described for a number of serotypes, including serotype 3 as suggested in other settings.^43^ The most recent published data from the UK report a non-significant vaccine effectiveness of 26% (95% CI: −69%, 68%), a very wide confidence interval crossing zero and a point estimate that is remarkably lower than the higher (and significant) effectiveness estimates obtained for the other PCV13 vaccine serotypes.^34^ Similarly, in the US, the effectiveness for PCV13 for serotype 3 was lower and the corresponding confidence intervals were large: 79.5% (95% CI: 30.3, 94.8).^44^

## Conclusion

Considering the recent body of evidence pointing to significant cross-protection for PHiD-CV against serotypes 6A and 19A as well as the limited protection for PCV13 against serotype 3, the comparison between the 2 vaccines for IPD essentially comes down to a 12-valent versus 12-valent discussion. This rationale also implies a similar efficacy for both vaccines against all-cause pneumonia when taking into account evidence showing that higher-valent vaccines do not automatically infer higher protection again pneumonia.^21,22^ Given the significant burden of AOM in children, the evidence of a positive impact for PHiD-CV against AOM caused by NT*Hi* coupled with a trend of pathogen replacement in AOM cases following the introduction of PCVs should tilt the balance of protection in children toward PHiD-CV.

Finally, it is interesting to note that the same lead-author was part of a recently published critical assessment of economic evaluations involving PHiD-CV or PCV13, which concluded “*the pivotal assumptions and results of these analyses also depended on which manufacturer sponsored the study*.”^45^ Wu et al. explained that this was due to “*fundamental uncertainties on serotype replacement and herd effects, serotype cross-protection and NTHi AOM protection*.”^45^ The challenges around the uncertainties around parameters used to populate economic models have been documented.^46^ According to International Society for Pharmacoeconomics and Outcomes Research's (ISPOR) guidance recommendations for conducting outcomes research, *“analysts should conform to evidence-based medicine principles (e.g., seek to incorporate all evidence, rather than selectively picking a single source; use best-practice methods to avoid potential biases, as when estimating treatment effectiveness from observational sources; employ formal evidence synthesis techniques)”* to estimate model parameters.^46^ Robustly applying the ISPOR guidelines for all analyses should minimize the discrepancies Wu et al. described.^45^ Importantly, we fully concur with Wu et al.'s final conclusions that “*decision makers using these analyses should not just rely on an analysis from a single manufacturer*” and would actively encourage all decision makers to rigorously evaluate the underlying assumptions used in all cost effectiveness analyses, irrespective of their source.^45^

## Trademark disclosure

Synflorix is a trademark of the GSK group of companies. Prevenar 13 is a trademark of Wyeth LLC.

## Abbreviations

11Pn-PD: 11-valent pneumococcal protein D conjugate vaccine
ACTIV: Association Clinique Thérapeutique Infantile du Val de Marne
CI: confidence interval
DTP-Hib: diphtheria, tetanus, pertussis-*Haemophilus influenzae* type B
ED: ear discharge
FinIP: Finnish Invasive Pneumococcal disease
FinOM: Finnish Otitis Media
IPD: invasive pneumococcal disease
IRR: Incidence Rate Ratio
ISPOR: International Society for Pharmacoeconomics and Outcomes Research's
JCVI: Joint Committee on Vaccines and Immunization
MEF: middle-ear fluid
NIP: national immunization program
NPC: nasopharyngeal carriage
NT*Hi* AOM: non-typeable *Haemophilus influenzae* acute otitis media
OPA: opsonophagocytic antibody
PCV: pneumococcal conjugate vaccine
PCV7: 7-valent pneumococcal conjugate vaccine
PCV13: 13-valent pneumococcal conjugate vaccine
PHiD-CV or PCV10: 10-valent pneumococcal non-typeable *Haemophilus influenzae* protein D conjugate vaccine
RCT: randomized control trial
RR: relative risk
UK: United Kingdom
US: United States
VT: vaccine-type
